# Identification of HOXB9 based on comprehensive bioinformatics analysis for predicting prognosis of head and neck squamous cell carcinoma

**DOI:** 10.1097/MD.0000000000035035

**Published:** 2023-09-01

**Authors:** Juanjuan Li, Hong Ran, Xiaoxia Zeng, Dunhui Yang, Xianhai Zeng, Peng Zhang

**Affiliations:** a Department of Otorhinolaryngology, Longgang Otorhinolaryngology Hospital & Shenzhen Key Laboratory of Otorhinolaryngology, Shenzhen Institute of Otorhinolaryngology, Shenzhen, Guangdong, China; b Department of Graduate and Scientific Research, Zunyi Medical University Zhuhai Campus, Zhuhai, Guangdong, China.

**Keywords:** bioinformatics, biomarker, head and neck cancer, immunology, prognosis

## Abstract

To evaluate the correlation between HOXB9 expression, and the prognosis and immune infiltration in head and neck squamous cell carcinoma (HNSCC). Pan-cancer HOXB9 expression was analyzed through TIMER2.0. The HOXB9 expression data of HNSCC and normal tissues were compared using the gene expression profiling interactive analysis (GEPIA) and the cancer genome atlas (TCGA) databases. The University of Alabama at Birmingham (UALCAN) database was used to analyze the relative expression of HOXB9 in HNSCC subgroups based on clinicopathological features, including cancer stage, tumor grade and lymph node stage. Survival analysis was performed using GEPIA, TCGA-Portal, Kaplan–Meier Plotter, and UALCAN databases. The genes co-expressed with HOXB9 were identified using TCGA data, and functionally annotated by GO and KEGG analyses. Protein-protein interaction network was constructed using the STRING database and Cytoscape 3.7.1. Single-sample gene set enrichment analysis was performed to assess the correlation between HOXB9 and immune infiltration based on TCGA data. TIMER 2.0 database was used to explore the correlation between HOXB9 expression and immune infiltration multiple cancers. HOXB9 mRNA is elevated in multiple cancers, and was upregulated in HNSCC tissues compared to non-paired (*P* < .05 in GEPIA; *P* < .0001 in TCGA) as well as paired (*P* < .0001 in TCGA) normal tissues. In addition, HOXB9 expression was positively correlated with tumor malignancy in the GEPIA and UALCAN databases (*P* < .05), and negatively with patient prognosis in both databases (*P* < .05). High HOXB9 expression was associated with increased infiltration of aDCs, NK CD56^bright^ cells, NK cells, and Th2 cells (*P* < .05), while low HOXB9 expression was associated with an increase in the proportion of DCs, iDCs, mast cells, neutrophils, and Th17 cells (*P* < .05). HOXB9 likely functions as an oncogene in HNSCC by disrupting the immune landscape, and is a promising prognostic biomarker and therapeutic target.

## 1. Introduction

Most head and neck cancers are derived from the mucosal epithelium in the oral cavity, pharynx, and larynx, and are known collectively as head and neck squamous cell carcinoma (HNSCC).^[1]^ It is the most common malignancy of the head and neck,^[1]^ and the 6th most common cancer worldwide, with 890,000 new cases and 450,000 deaths recorded in 2018.^[2]^ The conventional treatment strategies for HNSCC are associated with substantial morbidity and toxicity, and recurrent and metastatic disease is usually incurable. Currently, cetuximab is the only targeted drug approved for treating HNSCC.^[3]^ Therefore, it is critical to identify novel biomarkers for HNSCC in order to develop more effective therapies for the advanced disease.

The HOXB9 gene is a member of the Abd-B homeobox family and encodes a protein with a homeobox DNA-binding domain. It is part of a cluster of homeobox B genes located on chromosome 17. HOXB9 plays a key role in embryonic development as well as cancer progression.^[4–6]^ Silencing of HOXB9 in prostate cancer cells suppressed cellular proliferation, angiogenesis, migration and invasion.^[7]^ Furthermore, HOXB9 is associated with increased tumor angiogenesis and poor prognosis in patients with colon cancer, and HOXB9 expression has been positively correlated with gastric cancer progression and lymphangiogenesis.^[8]^ HOXB9 is also upregulated in endometrial cancer, and its elevated expression is associated with advanced histological grade, lymph node metastasis, and poor prognosis.^[9]^ Therefore, HOXB9 is a potential therapeutic target in cancer, although its regulatory signaling pathways in HNSCC remain elusive.

The aim of this study was to assess the biological role of HOXB9 in HNSCC. To this end, we systematically analyzed the expression, regulatory network, and prognostic relevance of HOXB9 in HNSCC. The correlation between tumor-infiltrating immune cells and HOXB9 expression was also analyzed. Overall, our findings suggest that high expression of HOXB9 portends poor prognosis and a dysregulated immune landscape in HNSCC. Thus, HOXB9 warrants further investigation as a potential prognostic biomarker and therapeutic target in HNSCC.

## 2. Materials and Methods

### 2.1. HOXB9 expression analysis

Pan-cancer HOXB9 mRNA expression data were downloaded from the cancer genome atlas (TCGA) database (n = 15776). The HOXB9 transcripts per million in the tumor tissues and corresponding normal tissues were analyzed using TIMER 2.0 (https://cistrome.shinyapps.io/timer/).^[10,11]^ The threshold for significant difference was |log2(fold-change) |> 1 and false discovery rate < 0.05.

HOXB9 mRNA levels in HNSCC tissues were analyzed through the gene expression profiling interactive analysis (GEPIA)-2 and xiantao web, and compared to that in normal tissues from the genotype-tissue expression records. The threshold for significant difference was log2 FC (log2 fold change) ≥ 1 or ≤ −1 and *P* value ≤ .01. The correlation between HOXB9 and the pathological stage was analyzed using the “Stage Plot” of GEPIA2 (http://gepia2.cancer-pku.cn/)^[12]^ and University of Alabama at Birmingham (UALCAN) databases (http://ualcan.path.uab.edu/).^[13]^

### 2.2. Survival analysis

The “Survival Map” component of GEPIA2 and UALCAN was used to compare the overall survival (OS) of HOXB9^high^ and HOXB9^low^ groups across different cancers in TCGA. The cutoff for distinguishing between the high- and low-expression groups was 50%. Survival curves were acquired from GEPIA2, and compared by the log-rank test.

### 2.3. Functional annotation of the co-expressed genes

GO and KEGG pathway enrichment analyses were performed to explore the functions of the genes co-expressed with HOXB9. A protein-protein interaction (PPI) network with a protein-protein confidence score ≥ 0.4 was established using the STRING database. Cytoscape software 3.7.1 (https://cytoscape.org/release_notes_3_7_1.html) was utilized to visualize the network. The correlation between HOXB9 and each of the top 9 hub genes was predicted by TIMER 2.0 and GEPIA2 database.

### 2.4. Immune cell infiltration analysis

The “Immune-Gene” unit of TIMER2 was used to assess the correlation between HOXB9 expression and immune infiltration in different cancers. The abundance of different immune cells was estimated using the QUANTISEQ, XCELL, MCPCOUNTER, TIMER, CIBERSORT, and CIBERSORT-ABS algorithms. *P* values and partial correlation (cor) values were determined by Spearman rank correlation test, purity-adjusted. The outcomes were displayed in the form of a scatterplot and a heatmap.

### 2.5. Statistical analysis

*P* < .05 was considered statistically significant. The analysis and visualization of the data were done by stated online databases (GEPIA2, UALCAN, TIMER2.0, and Xiantao web) and tools (Cytoscape 3.7.1).

## 3. Results

### 3.1. HOXB9 is upregulated in multiple cancer tissues

**Figure 1.**
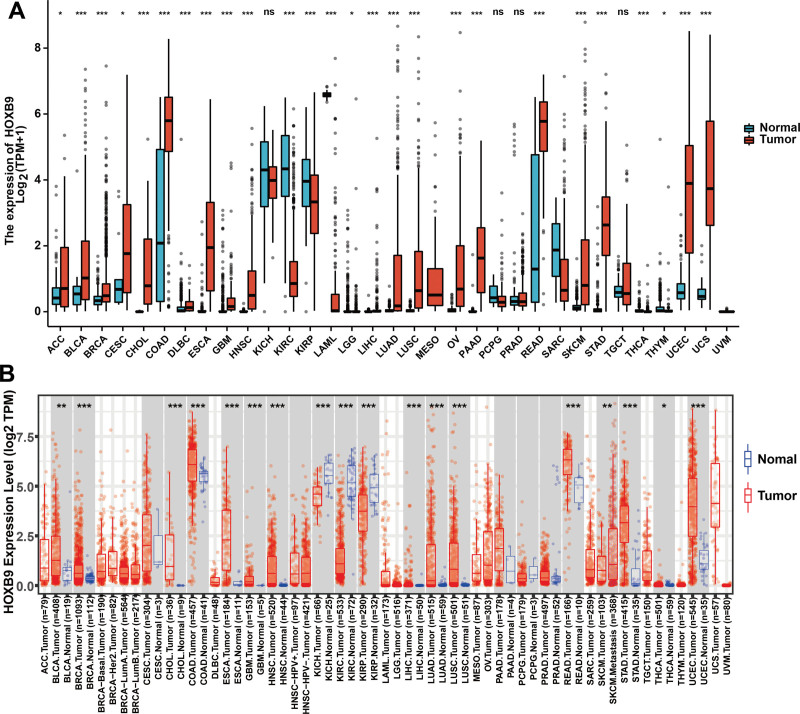
Pan-cancer HOXB9 mRNA expression. (A) HOXB9 expression in TCGA database. (B) HOXB9 expression in TIMER 2.0 database. (*: *P* value < 0.05; **: *P* value < 0.01; ***: *P* value < 0.001). TCGA = the cancer genome atlas.

As shown in Figure 1A, HOXB9 mRNA levels were significantly higher in HNSCC, BRCA, COAD, ESCA, LIHC, LUAD, LUSC, READ, STAD, and THCA samples in TCGA datasets. In contrast, HOXB9 was downregulated in KIRC, KIRP and SKCM. Further analysis of 31 tumor types with TIMER2.0 indicated that HOXB9 expression was significantly higher in HNSCC, COAD, ESCA, READ, STAD, and UCS, and lower in KIRC and LAML (Fig. 1B). Thus, the results from both databases were consistent.

### 3.2. Elevated HOXB9 is associated with increased malignancy of HNSCC tumors

**Figure 2.**
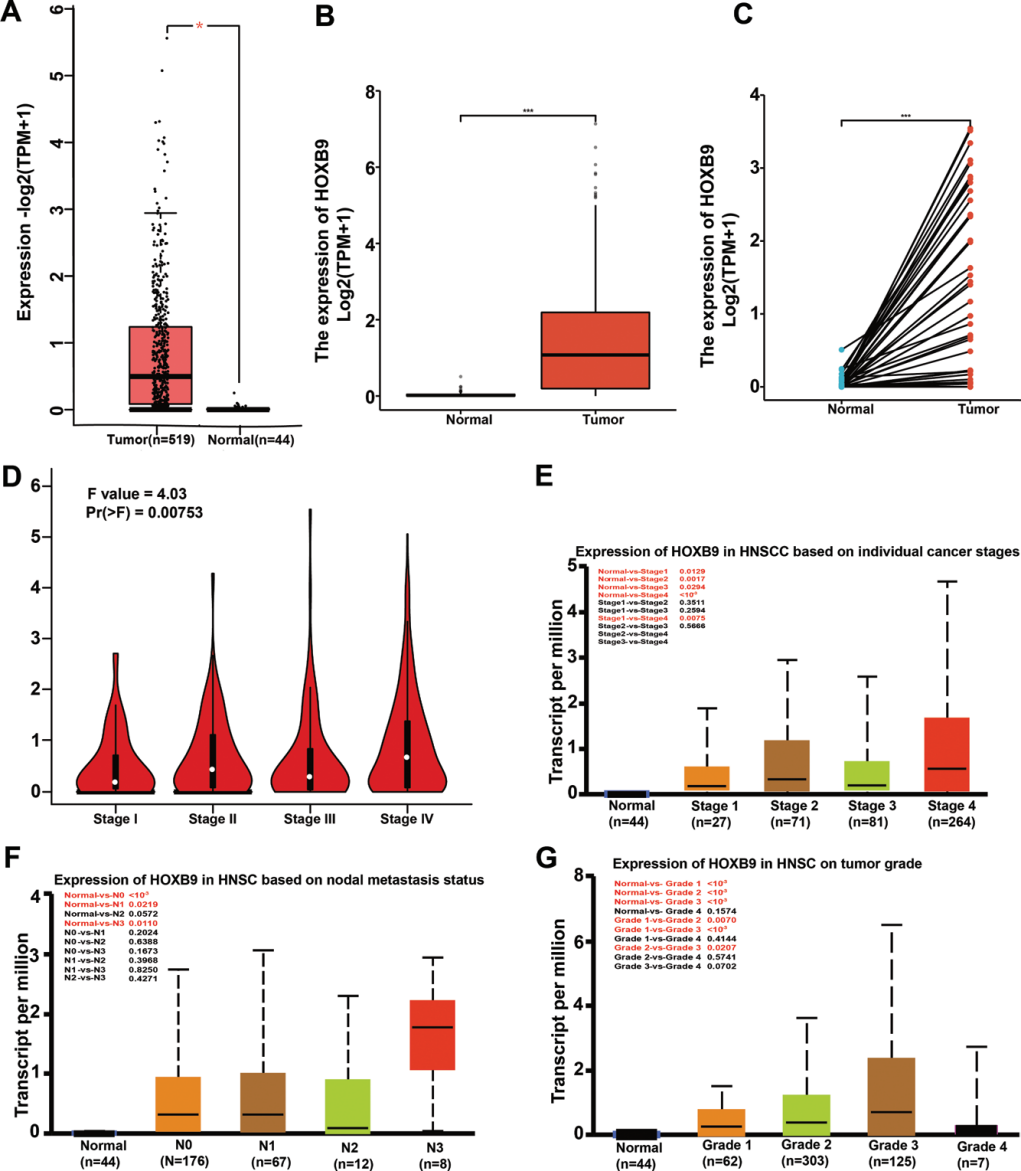
HOXB9 was upregulated in HNSCC and correlated with tumor progression. (A and B) Expression of HOXB9 gene in tumor and normal tissues in the UALCAN and GEPIA2 databases, (C) HOXB9 expression in HNSCC and paired non-tumor tissues, (D–G) HOXB9 expression in the different (D) pathological stages (I, II, III, and IV), (E) tumor stages (T1, T2, T3, and T4), (F) lymph node stages (N0 1, 2, and 3), and (G) tumor grades (N0, 1, 2, 3, and 4). (*: *P* value < 0.05; **: *P* value < 0.01; ***: *P* value < 0.001). GEPIA = gene expression profiling interactive analysis, HNSCC = head and neck squamous cell carcinoma, UALCAN = University of Alabama at Birmingham.

Assessment of the GEPIA2 database consisting of 619 HNSCC and 44 adjacent normal tissue specimens demonstrated that HOXB9 mRNA levels were significantly higher in HNSCC compared to that in the normal tissues (Fig. 2A). Data from TCGA also confirmed the overexpression of HOXB9 in HNSCC (Fig. 2B and C). Furthermore, HOXB9 expression was strongly correlated with the tumor stage (Fig. 2D), and was significantly higher in the middle and advanced stages compared to the earlier stages, which suggests a potential function of HOXB9 in tumor development and metastasis (Fig. 2E). In addition, HOXB9 was upregulated in the lymph nodes at all stages of cancer development (Fig. 2F), and in the high-grade tumors compared to normal tissues (Fig. 2G). These findings indicated that HOXB9 is associated with the malignant behavior of HNSCC.

### 3.3. HOXB9 is a prognostic biomarker of HNSCC

**Figure 3.**
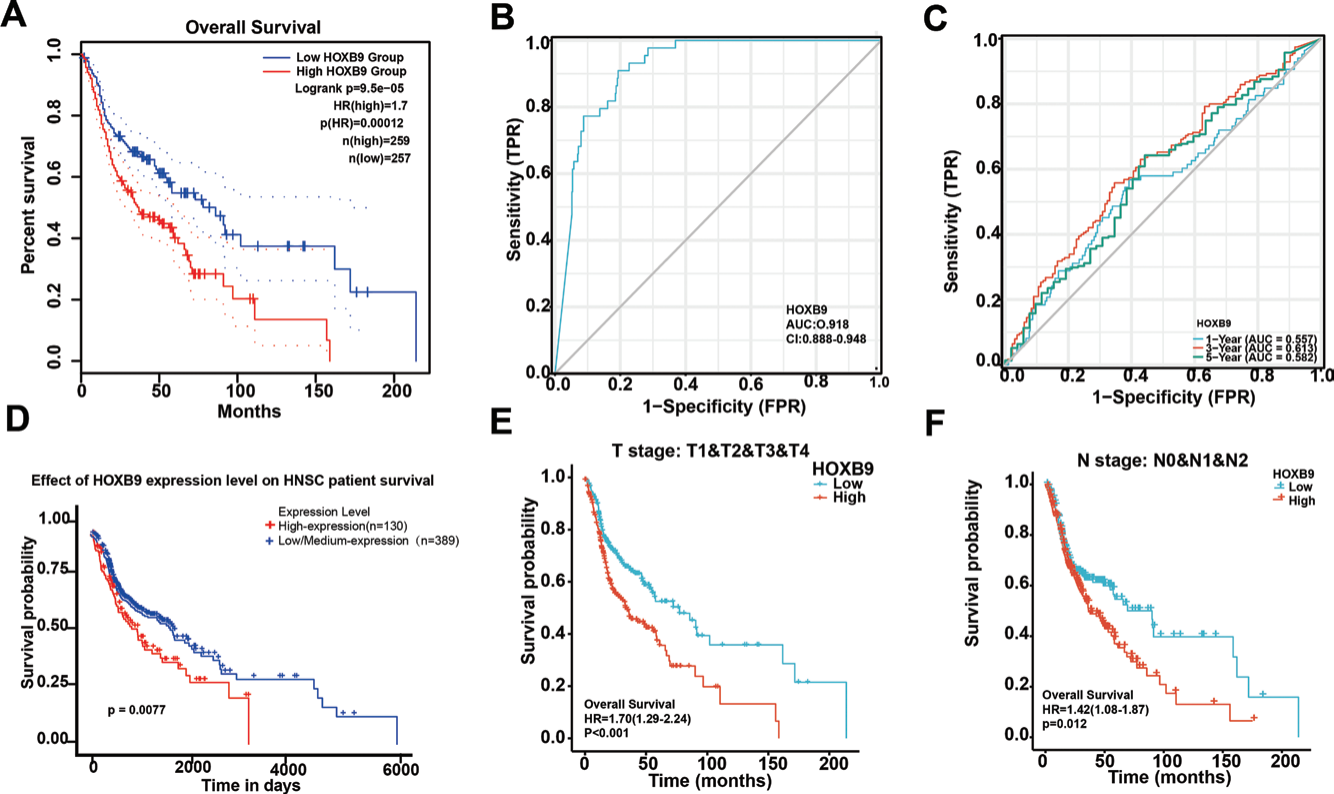
KM survival plots and ROC curves. (A) OS curves of HNSCC patients demarcated by HOXB9 expression in the GEPIA dataset. (B) ROC curve for the prognostic value of HOXB9. (C) Time-dependent ROC curves showing the predictive ability of HOXB9 for 1-, 3-, and 5-year OS. (D) OS curves of HOXB9^high^ and HOXB9^low^ HNSCC patients in the UALCAN dataset. (E and F) Subgroup analysis for tumor stage (T1/T2/T3/T4) and lymph node stage (N0/N1/N2). GEPIA = gene expression profiling interactive analysis, HNSCC = head and neck squamous cell carcinoma, OS = overall survival, ROC = receiver operating characteristic, UALCAN = University of Alabama at Birmingham.

HNSCC patients with higher expression of HOXB9 had significantly worse prognosis compared to the HOXB9^low^ group (Fig. 3A, *P* < .05). The predictive power of HOXB9 was evaluated by receiver operating characteristic curve analysis, and the area under the curve was calculated. As shown in Figure 3B, the area under the curve of HOXB9 for predicting the prognosis of HNSCC patients was 0.918, and that for 1-, 3-, and 5-year survival probabilities were 0.557, 0.613, and 0.582 respectively. Thus, HOXB9 can better predict the probability of OS within 3 years rather than the long-term survival (Fig. 3C). In the ULCAN dataset as well, elevated HOXB9 expression was highly correlated with poor prognosis (Fig. 3D), tumor malignancy (*P* < .001) and lymph node transformation (*P* = .012) (Fig. 3E and F). Taken together, elevated HOXB9 expression is strongly associated with poor prognosis in HNSCC, and is an effective prognostic biomarker.

### 3.4. Functional analysis of HOXB9-associated co-expressed genes

**Figure 4.**
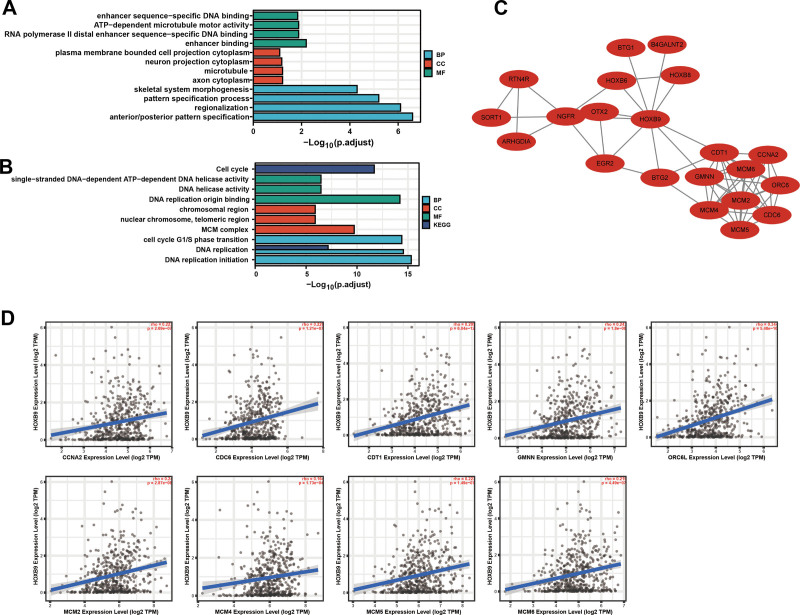
Results of GO and KEGG analyses. (A) Results of GO enrichment analysis. (B) Results of KEGG enrichment analysis. (C) The PPI network based on genes co-expressed with HOXB9. (D) Correlation between HOXB9 expression and the top 9 hub genes in the TIMER 2.0 database. PPI = Protein-protein interaction.

To further explore the biological role of HOXB9 in HNSCC, we functionally annotated the genes co-expressed with HOXB9 through GO and KEGG pathway enrichment analyses. As shown in Figure 4A, the HOXB9-interacting genes were mainly enriched in biological processes including anterior/posterior pattern specification, regionalization, and pattern specification process. In addition, KEGG pathway analysis showed that these genes were enriched in pathways associated with DNA repair or signal transduction such as cell cycle, DNA replication origin binding, and DNA replication intitiation (Fig. 4B). Based on TCGA database, we chose the top 21 co-expressed genes to construct a PPI network using STRING and Cytoscape software. The top 9 genes with the highest connectivity were CCNA, CDC6, CDT1, GMNN, ORC6L, MCM2, MCM4, MCM5, and MCM6 (Fig. 4C). As shown in Figure 4D, HOXB9 expression was correlated to that of CCNA (rho = 0.223, *P* = 2.69e–0.7), CDC6 (rho = 0. 229, *P* = 1.21e–07), CDT1 (rho = 0.295, *P* = 6.04e–12), GMNN (rho = 0.243, *P* = 1.8e–8), ORC6L (rho = 0.344, *P* = 5.58e–16), MCM2 (rho = 0. 24, *P* = 2.87e–08), MCM4 (rho = 0.164, *P* = 1.73e–04), MCM5 (rho = 0.227, *P* = 1.49e–07), MCM6 (rho = 0.219, *P* = 4.49e–07).

### 3.5. Correlation between HOXB9 and immune cell infiltration

**Figure 5.**
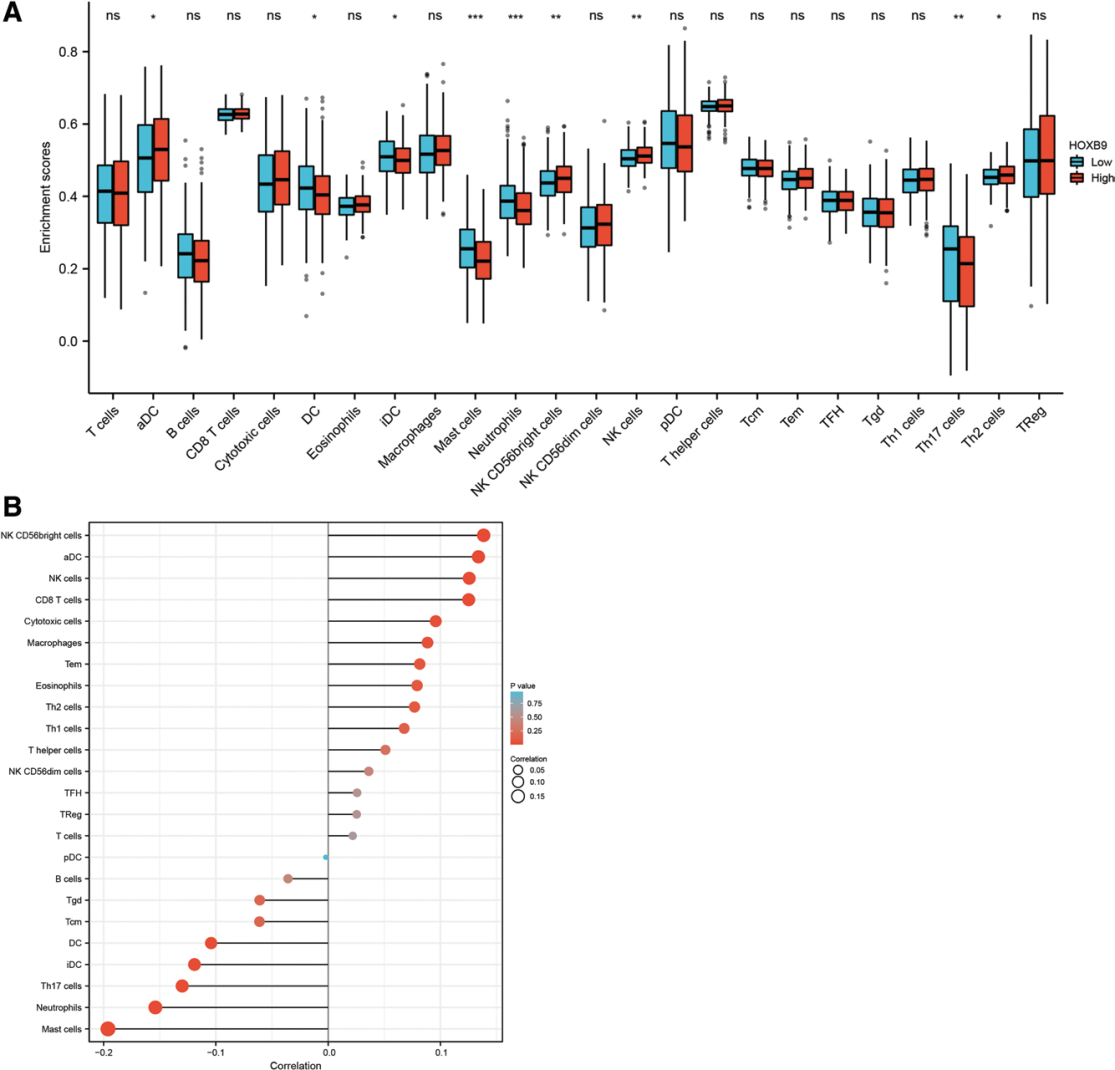
HOXB9 is associated with the immune landscape of HNSCC. (A) Analysis of the proportion of immune cells from ssGSEA. (B) The association between HOXB9 and immune cells in HNSCC in TCGA dataset. ns: no significance, *P < .05, **P < .01, ***P < .001. HNSCC = head and neck squamous cell carcinoma, ssGSEA = single-sample gene set enrichment analysis, TCGA = the cancer genome atlas.

**Figure 6.**
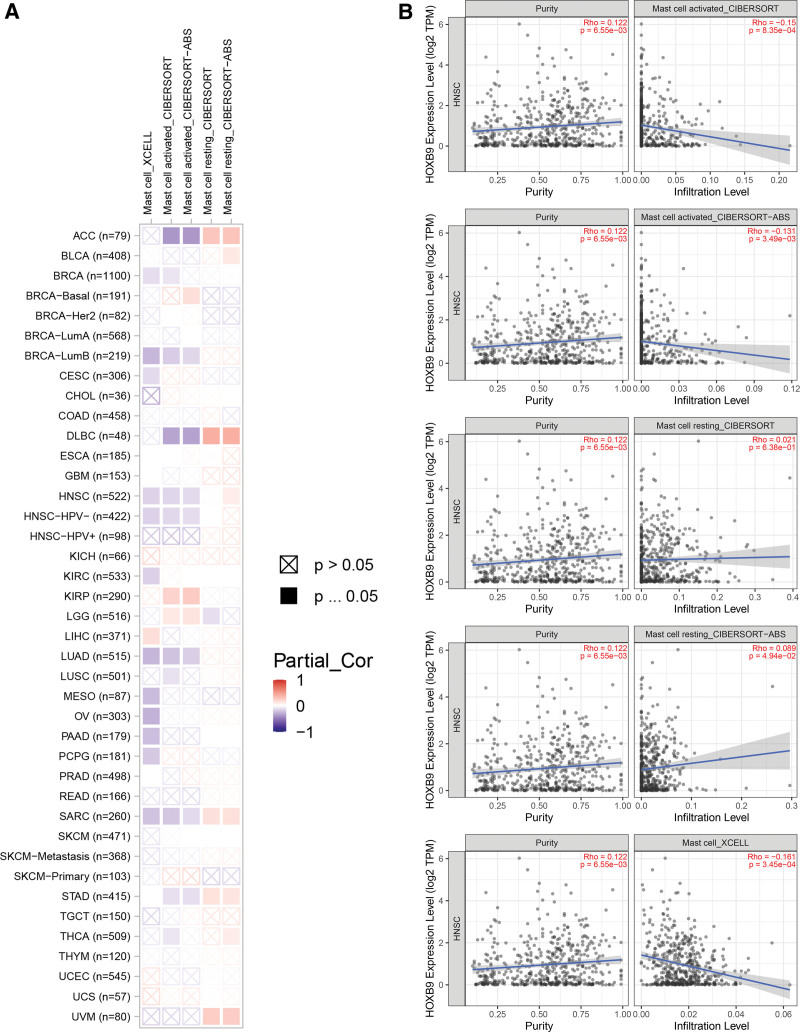
Association between HOXB9 expression and infiltration of mast cells in various cancer types using the XCELL, CIBERSORT, and CIBERSORT-ABS algorithms.

Immune cells are among the major components of the tumor microenvironment and play a crucial part in tumor initiation, development, and metastasis.^[11]^ Therefore, we further evaluated the immune landscape in the HOXB9^high^ and HOXB9^low^ groups using 546 HNSCC sample from TCGA. As shown in Figure 5A, HOXB9 overexpression was associated with an increased abundance of aDCs, NK CD56^bright^ cells, NK cells, and Th2 cells (*P* < .05), and lower abundance of DCs, iDCs, mast cells, neutrophils and Th17 cells. According to the results of single-sample gene set enrichment analysis, HOXB9 expression was correlated with the number of various innate immune cells, of which the mast cells showed the strongest correlation (Fig. 5B). Similar correlation was observed between the grade of immune infiltration and HOXB9 expression across different cancer types in TCGA database with the XCELL, CIBERSORT and CIBERSORT-ABS algorithms, and the results revealed a positive correlation between HOXB9 and infiltration of mast cells in HNSCC (Fig. 6).

## 4. Discussion

The transcriptomic data of TCGA has been instrumental in unearthing the molecular mechanisms underlying cancer progression, and in the identification of novel therapeutic and diagnostic biomarkers for various cancers.^[12–14]^ In addition, multiple public resources such as cBioPortal,^[15]^ miRGator v 3.0,^[16]^ and TANRIC^[17]^ have facilitated the comprehensive analysis of TCGA transcriptome data, and user-friendly interfaces like UALCAN and GEPIA allow the identification of survival associations involving any gene of interest across different cancer types and pathological subtypes.^[18]^

Despite advances in the diagnosis and treatment of HNSCC over the past few decades, the prognosis for patients with advanced disease remains poor. Immunotherapy has emerged as an alternative, and several immune checkpoint inhibitors have improved survival rates of patients with advanced HNSCC.^[3,19–21]^ However, a significant number of patients gradually become unresponsive to immunotherapy due to development of treatment resistance. Therefore, it is crucial to explore sensitive diagnostic and prognostic biomarkers for HNSCC.

HOXB9 plays a critical role in many human solid cancers and its aberrant expression drives tumor formation.^[22]^ High levels of HOXB9 have been associated with poor prognosis in lung adenocarcinoma,^[23]^ poor OS in colon cancer,^[24]^ high cancer grade and poor OS in breast cancer,^[25]^ and vascular infiltration and poor OS in hepatocellular carcinoma.^[5]^ In our pan-cancer analysis, we found that HOXB9 expression was elevated in various tumor types with broadly similar prognostic results. Likewise, HOXB9 was highly expressed in HNSCC tissues and correlated positively with poor prognosis. Recent studies have shown that multiple biological processes such as signaling, regulation of gene expression, energy metabolism, and cell cycle regulation are dependent on PPI networks and not just individual proteins.^[26]^ In our study, CCNA, CDC6, CDT1, GMNN, ORC6L, MCM2, MCM4, MCM5, and MCM6 were identified as the hub genes co-expressed with HOXB9, and these genes are enriched in DNA repair or signal transduction, such as cell cycle, DNA replication origin binding and DNA replication initiation.

Mast cells can reshape the immune microenvironment of tumors and greatly influence tumorigenesis and progression.^[27]^ Few studies have focused on the mast cell signature genes of HNSCC, particularly their potential mechanisms. One study established a mast cell gene-based signature for HNSCC to predict the prognosis and immunotherapeutic response.^[28]^ Consistent with these previous reports, we found that HOXB9 expression was positively correlated to the infiltration of mast cells, indicating that the prognostic differences between the HOXB9^high^ and HOXB9^low^ groups can be attributed to immune regulation.

Our study has several limitations that ought to be considered. First, this study was based on a public database, and the findings will have to be validated using external samples. Second, inclusion of normal tissues as control in the receiver operating characteristic analysis could provide more convincing evidence that HOXB9 is a potential diagnostic and therapeutic marker of HNSCC. Third, the biological functions of HOXB9 in HNSCC need to be investigated further using cell lines and animal models. Overall, our findings indicate that HOXB9 is a potential target for HNSCC immunotherapy and a reliable prognostic biomarker.

## Author contributions

**Data curation:** Juanjuan Li, Hong Ran.

**Formal analysis:** Juanjuan Li, Hong Ran, Xiaoxia Zeng, Dunhui Yang.

**Funding acquisition:** Xiaoxia Zeng, Xianhai Zeng, Peng Zhang.

**Investigation:** Juanjuan Li, Hong Ran, Xiaoxia Zeng, Dunhui Yang.

**Methodology:** Juanjuan Li, Hong Ran.

**Project administration:** Xianhai Zeng, Peng Zhang.

**Software:** Dunhui Yang.

**Supervision:** Xianhai Zeng, Peng Zhang.

**Validation:** Hong Ran, Dunhui Yang.

**Writing – original draft:** Juanjuan Li, Hong Ran, Xianhai Zeng, Peng Zhang.

**Writing – review & editing:** Xianhai Zeng, Peng Zhang.
